# Comparative Genome Analyses Reveal the Genomic Traits and Host Plant Adaptations of *Flavobacterium akiainvivens* IK-1^T^

**DOI:** 10.3390/ijms20194910

**Published:** 2019-10-03

**Authors:** Xuehua Wan

**Affiliations:** 1 TEDA Institute of Biological Sciences and Biotechnology, Nankai University, TEDA, Tianjin 300071, China; xuehua.wan@hotmail.com; 2 The Key Laboratory of Molecular Microbiology and Technology, Ministry of Education, Nankai University, Tianjin 300071, China; 3 Tianjin Key Laboratory of Microbial Functional Genomics, Nankai University, Tianjin 300071, China

**Keywords:** comparative genomics, pan genome, synteny, evolution direction, plant-associated bacteria

## Abstract

The genus *Flavobacterium* contains a large group of commensal bacteria identified in diverse terrestrial and aquatic habitats. We compared the genome of a new species *Flavobacterium akiainvivens* IK-1^T^ to public available genomes of *Flavobacterium* species to reveal the genomic traits and ecological roles of IK-1^T^. Principle component analysis (PCA) of carbohydrate-active enzyme classes suggests that IK-1^T^ belongs to a terrestrial clade of *Flavobacterium*. In addition, type 2 and type 9 secretion systems involved in bacteria-environment interactions were identified in the IK-1^T^ genome. The IK-1^T^ genome encodes eukaryotic-like domain containing proteins including ankyrin repeats, von Willebrand factor type A domain, and major royal jelly proteins, suggesting that IK-1^T^ may alter plant host physiology by secreting eukaryotic-like proteins that mimic host proteins. A novel two-component system *Fa*RpfC-*Fa*YpdB was identified in the IK-1^T^ genome, which may mediate quorum sensing to regulate global gene expressions. Our findings suggest that comparative genome analyses of *Flavobacterium* spp. reveal that IK-1^T^ has adapted to a terrestrial niche. Further functional characterizations of IK-1^T^ secreted proteins and their regulation systems will shed light on molecular basis of bacteria-plant interactions in environments.

## 1. Introduction

The genus *Flavobacterium* belongs to the phylum *Bacteroidetes* and has been known for almost one hundred years, which was proposed in the first edition of *Bergey’s Manual of Determinative Bacteriology* in 1923 [1]. More than 130 species have been identified in the genus *Flavobacterium*, which are isolated from terrestrial and aquatic environments. Three species, including *F. psychrophilum*, *F. columnare*, and *F. branchiophilum*, can cause severe fish diseases worldwide. However, most *Flavobacterium* spp. are commensal bacteria isolated with greater abundances from rivers and oceans [2]. Aquatic *Flavobacteria* played important roles in algae decomposition [2,3,4], while terrestrial *Flavobacteria* are adapted to plant carbohydrate metabolism and may promote plant growth and development [5,6]. 

Microorganisms are natural resources of chemicals such as biopigments and biosurfactants that have promising potentials in biotechnological and industrial applications. The genus *Flavobacterium* yields carotenoids, a huge family of pigments, displaying various colors, including yellow, orange, pink, red, and brown. Several species of *Flavobacterium* are able to degrade organic contaminants, such as pentachlorophenol, nylon oligomers, polyaromatics, and pesticides [7,8,9,10,11,12]. Screening for biosurfactant-producing microorganisms identified one *Flavobacterium* strain as a biosurfactant-producing microbe [13]. Structure analyses revealed the *Flavobacterium* strain produced at least 37 flavolipids as a new class of biosurfactants [7]. Identification and characterization of *Flavobacterium* species and strains may help reveal biotechnological applications of this genus.

*F. akiainvivens* IK-1^T^, isolated from decaying shrub *Wikstroemia oahuensis* in Hawaii, has been formally described with a validly published name [14]. IK-1^T^ was Gram-negative and non-motile, and its gliding motility was not observed [14]. A colony formed by IK-1^T^ showed translucent and off-white [14]. Based on the comparisons of the 16S rRNA gene sequences, the nearest phylogenetic neighbors of IK-1^T^ were *F. rivuli* WB 3.3-2^T^ and *F. subsaxonicum* WB 4.1-42^T^, which were isolated from a hard water rivulet of North Germany and characterized to be non-motile and non-gliding [14,15,16]. Analysis of the *F. rivuli* genome has revealed 151 genes encoding carbohydrate-active enzymes (CAZy) and 177 genes encoding peptidases, implying a role of *F. rivuli* in the decomposition potential of organic matter in the environment [15]. In addition, although *F. rivuli* was isolated from stream water, it was affiliated with genomes of terrestrial clade-affiliated *Flavobacteria* [5]. A fingerprinting-based study on flavobacterial community structure also supported that *F. rivuli* is soil-derived [17]. 

The assembled genome sequence of IK-1^T^ was briefly reported [18]. However, the genome has not been analyzed in details yet. In this study, we compared the IK-1^T^ genome to genomes of fifty-eight members of its related *Flavobacterium* species and characterized IK-1^T^ at a whole genome level. Our findings reveal that IK-1^T^ belongs to a terrestrial clade of *Flavobacteria* and has adapted to plant host.

## 2. Results

### 2.1. Genome Features of IK-1^T^

**Figure 1:**
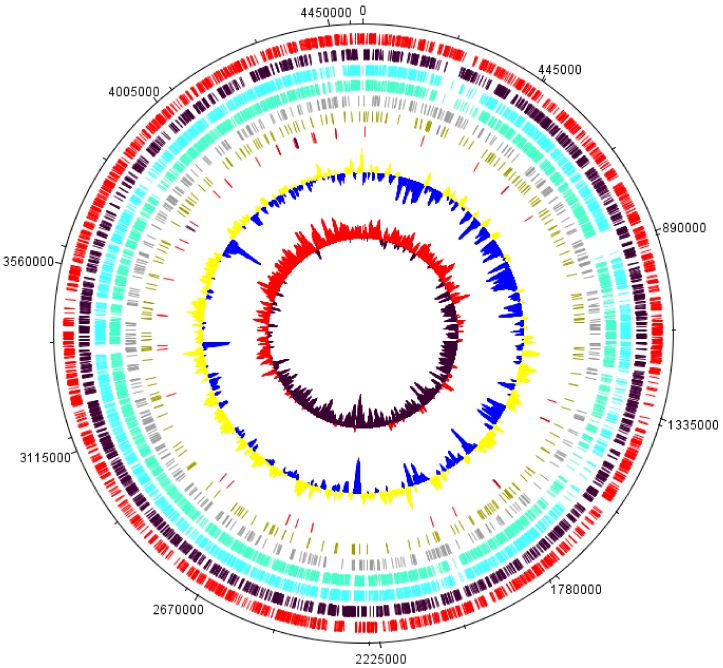
Genome features of *F. akiainvivens* IK-1^T^. From the outside to the center: 1. tick marks, 2. coding sequences in forward strand (red), 3. coding sequences in reverse strand (black), 4. IK-1^T^-*F. subsaxonicum* orthologs (light blue), 5. IK-1^T^-*F. rivuli* orthologs (green), 6. IK-1^T^ singleton (grey), 7. repeats (light green) and CRISPR (blue), 8. tRNA (red) and rRNA (black), 9. GC plot, below average (43.8%) (blue) and above average (yellow), 10. GC skew [(G − C)/(G + C)], below average (0.028%) (black) and above average (red).

The assembled genome sequence of IK-1^T^ contains a single chromosome spanning 4.5 Mb [18]. To provide deep analyses of this new species, we annotated the IK-1^T^ genome in details. Prokka predicted 50 tRNA, and 4067 protein-coding genes (Table S1). A total of 1978 proteins were annotated with functions, and 2089 proteins were annotated as hypothetical proteins with unknown functions. We further identified 20,351 bp of repeats in the genome using RepeatMasker (Table S2). Most of the repeats belong to simple repeats (9336 bp) and low complexity regions (2258 bp). The general genome features of IK-1^T^ are shown in Figure 1.

### 2.2. IK-1^T^ is a Member of Terrestrial Clade-Affiliated Flavobacteria

**Figure 2:**
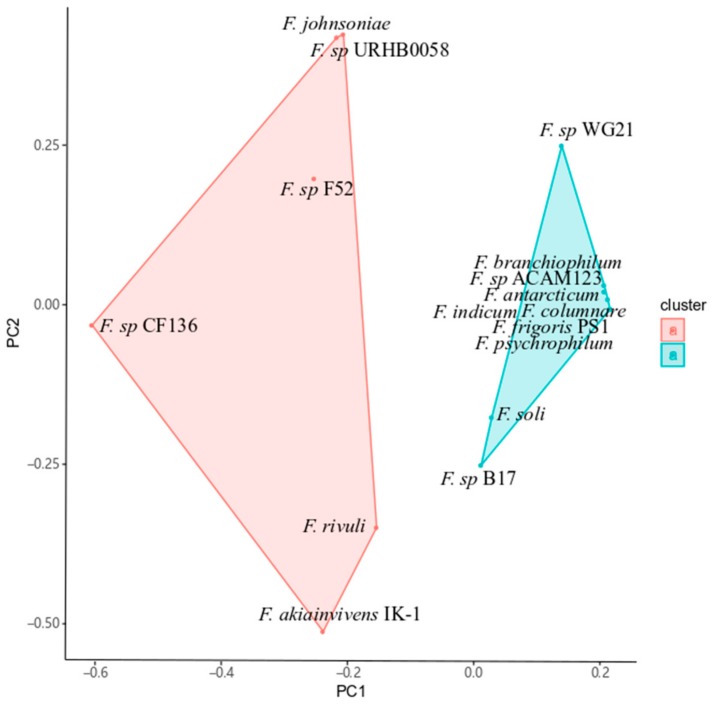
Two dimensional scatter plot based on carbohydrate-active enzyme (CAZy) classes in the terrestrial and aquatic flavobacteria. The matrix representing the presence and absence of the CAZy classes was imported into the R program. IK-1^T^ was clustered with members from the terrestrial clade of *Flavobacteria* (red). Members from the aquatic clade of *Flavobacteria* (blue) was clustered separately. The terrestrial and aquatic clades of *Flavobacteria* showed distinct distances in this two dimensional plot. The percentages of PC1 and PC2 are 84.9% and 8.49%, respectively. Red: terrestrial *flavobacteria*, blue: aquatic *flavobacteria*.

Comparative gene analysis suggested that the terrestrial and aquatic *Flavobacteria* can be hierarchically clustered into distinct clades [5]. The genomes of members in the terrestrial clade-affiliated *Flavobacteria* encode glycoside hydrolase (GH) families GH78 and GH106 for utilization of rhamnogalacturonan in plant cell wall [5]. To investigate whether IK-1^T^ is a terrestrial bacterium associated with plant, we identified the gene families of carbohydrate-active enzymes (CAZy) in the genome, according to the previously reported method [5]. We surveyed 16 CAZy families including GH, carbohydrate esterases (CE), polysaccharide lyases (PL), and carbohydrate-binding module (CBM). The abundance and distribution of CAZy families in IK-1^T^ indicated that it is a terrestrial bacterium, compared to those in seven terrestrial and eight aquatic *Flavobacterium* spp. (Table S3). We found that the IK-1^T^ genome encodes GH78 and GH106 genes, suggesting that IK-1^T^ belongs to the terrestrial clade of *Flavobacteria*. Likewise, 17 GH43 and two GH27 involved in metabolism of glucans containing arabinose were found in the IK-1^T^ genome, which are associated with the terrestrial clade-affiliated *Flavobacteria*. Principle component analysis (PCA) of the CAZy gene families further statistically clustered IK-1^T^ with the terrestrial *Flavobacterium* spp., indicating that IK-1^T^ has adapted to a terrestrial environment (Figure 2). 

### 2.3. An Open Pan-Genome of Flavobacteria

We downloaded the genome sequences of 58 members of the genus *Flavobacterium* and re-annotated them using Prokka. The detailed information was summarized in Supplementary Table S4. A total of 215,727 protein sequences were loaded for all-vs.-all BLASTP. We removed any alignments at or below 80% of aligned length and 30% identity. We performed OrthoMCL analysis to identify 13,583 orthologous groups (Table S5). Only seven orthologous groups were conserved in the 59 genomes of *Flavobacterium* species. Because the genomes of three strains (SCGC AAA536-P05, MS220-5C, and JGI 0001001-D01) contained fewer than 2000 genes, we excluded them and found that 541 orthologous groups were conserved in the 56 genomes of *Flavobacteria*. These results indicated that gene gain-and-loss events occurred in *Flavobacteria*, which contribute to the genome dynamics and biodiversity of *Flavobacteria*. 

**Figure 3:**
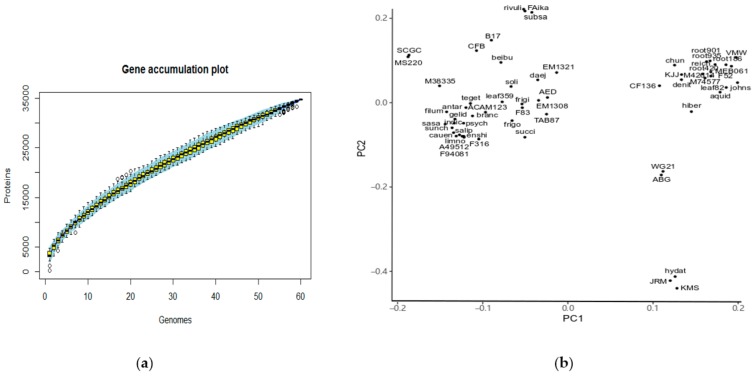
Pan-genome of *Flavobacteria*. (**a**) Gene accumulation plot of pan-genome of *Flavobacteria*. Each box represents the values for any possible combination of gene clusters in the respective number of genomes. (**b**) Two dimensional scatter plot based on the presence and absence of gene families in the *Flavobacteria* genus. The binary matrix representing the presence and absence of gene families was imported into the R program. IK-1^T^ showed close relationship to *F. rivuli* and *F. subsaxonicum*, and showed distinct distances to other species in the *Flavobacterium* genus. The percentages of PC1 and PC2 in Figure 3b are 15.95% and 4.88%, respectively.

The pan-genome of *Flavobacteria* contained 34,769 genes (Figure 3a). By applying power law regression using gene number medians, the gene accumulation curve fitted Heaps’ law and indicated an open pan-genome which is far from saturation (α = 0.57, R^2^ = 0.998) [19,20]. We recorded the presence and absence of the orthologous groups in a binary matrix. PCA analysis of the presence and absence of the orthologous groups showed close relationships between IK-1^T^ and *F. rivuli*/*F. subsaxonicum* (Figure 3b), consistent with the comparison result of 16S rRNA gene sequences. Likewise, phylogenetic analysis using concatenated single-ortholog groups also clustered IK-1^T^ with *F. rivuli* and *F. subsaxonicum* (Figure S1). These data suggest that *Flavobacteria* occupy diversified niches that lead to an open and dynamic pan-genome. In addition, the nearest phylogenetic neighbors of IK-1^T^ are *F. rivuli* and *F. subsaxonicum*. 

To reveal species-specific genes in IK-1^T^, orthoMCL predicted 596 singletons in the IK-1^T^ genome, and 491 singletons (82.4%) were annotated as hypothetical proteins. The 105 singletons were annotated with functions including hydrolases, proteases, transferases, oxidoreductases, histidine kinases, transcriptional regulators/activators, transmembrane proteins, and transporters. The singletons were re-annotated using InterProScan and plotted for Gene Ontology (GO) annotation (Figure S2). These IK-1^T^-specific genes contribute to the large size of the open pan-genome of *Flavobacteria*.

### 2.4. Synteny Between IK-1^T^ and F. rivuli/F. subsaxonicum

**Figure 4:**
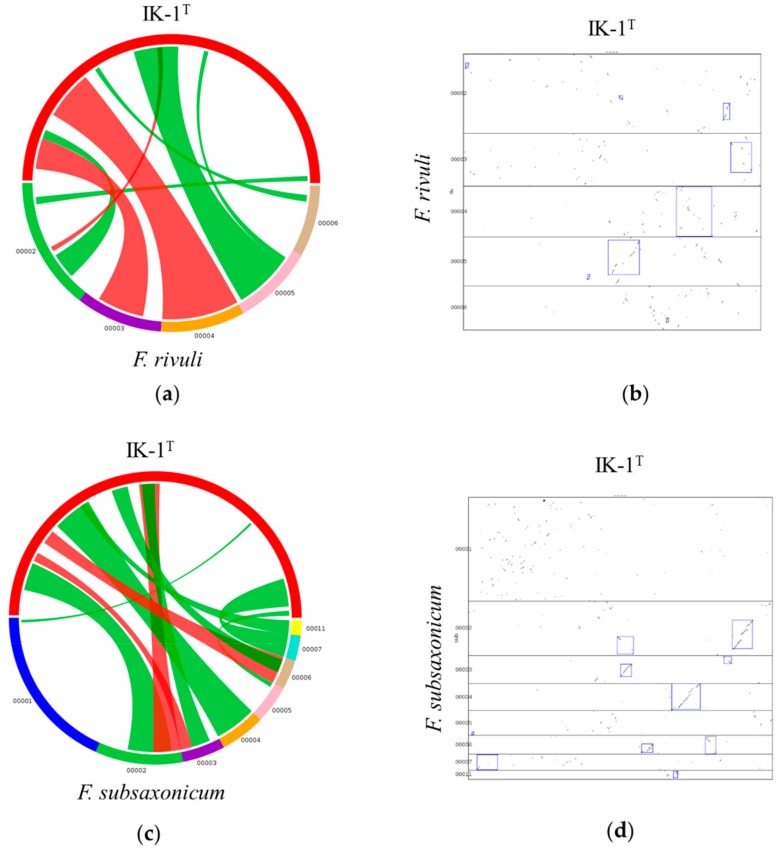
Syntenic blocks shared between IK-1^T^ and its nearest phylogenetic neighbors. (**a**) Syntenic blocks shared between IK-1^T^ and *F. rivuli*. (**b**) Syntenic regions shared between IK-1^T^ and *F. rivuli* visualized by dotplot. (**c**) Syntenic blocks shared between IK-1^T^ and *F. subsaxonicum*. (**d**) Syntenic regions shared between IK-1^T^ and *F. subsaxonicum* visualized by dotplot. Red: forward synteny, green: reverse synteny. Red in the circle: IK-1^T^, other colors in the circle: scaffolds of *F. rivuli* (**a**) or *F. subsaxonicum* (**c**).

Genome rearrangement and synteny provide evolutionary relationships between genomes. We used SyMAP to determine syntenic blocks in IK-1^T^-*F. rivuli* and IK-1^T^-*F. subsaxonicum*. Comparison of the genomes of IK-1^T^ and *F. rivuli* revealed eight syntenic blocks, while comparison of the genomes of IK-1^T^ and *F. subsaxonicum* revealed eleven syntenic blocks, spanning 1,614,043 bp (36%) and 2,026,781 bp (45%) in the IK-1^T^ genome, respectively (Figure 4a,c, Table S6). Synteny blocks and dot plots were shown in Figure 4b and d. Majority of the genome regions were not in the syntenic blocks. By contrast, comparison of the genomes of *F. rivuli* and *F. subsaxonicum* revealed sixty-eight syntenic blocks, covering 74% and 75% of the genome sequences of *F. rivuli* and *F. subsaxonicum*, respectively (Figures S3 and S4). These findings suggest that the level of synteny between *F. rivuli* and *F. subsaxonicum* is much higher than that between IK-1^T^ and *F. rivuli*/*F. subsaxonicum*. In addition, we compared the genomes of IK-1^T^ and an outgroup member *Elizabethkingia meningoseptica* for synteny analysis. *E. meningoseptica* was originally described as *Flavobacterium meningoseptica*. Both *Elizabethkingia* and *Flavobacterium* belong to the Family *Flavobacteriaceae*. The genome of *E*. *meningoseptica* (GenBank: CP016378.1) contains 4 Mb sequences and encodes 3647 genes. Only one syntenic block was identified between the genomes of *E*. *meningoseptica* (2%) and IK-1^T^ (5%) (Figures S5 and S6). These data suggest that extreme low level of synteny was found between IK-1^T^ and the outgroup member. Taken together, the above data suggest that dramatic rearrangements occurred in the genomes of IK-1^T^ and *F. rivuli* or *F. subsaxonicum* after they diverged from their most recent common ancestor (MRCA). Moreover, extensive synteny and conserved gene orders exist between these genomes. This synteny may be due to incomplete dislocation of gene order or selective aggregation of genes under evolutionary pressures.

### 2.5. Evolution Direction in the IK-1^T^ Genome

**Figure 5:**
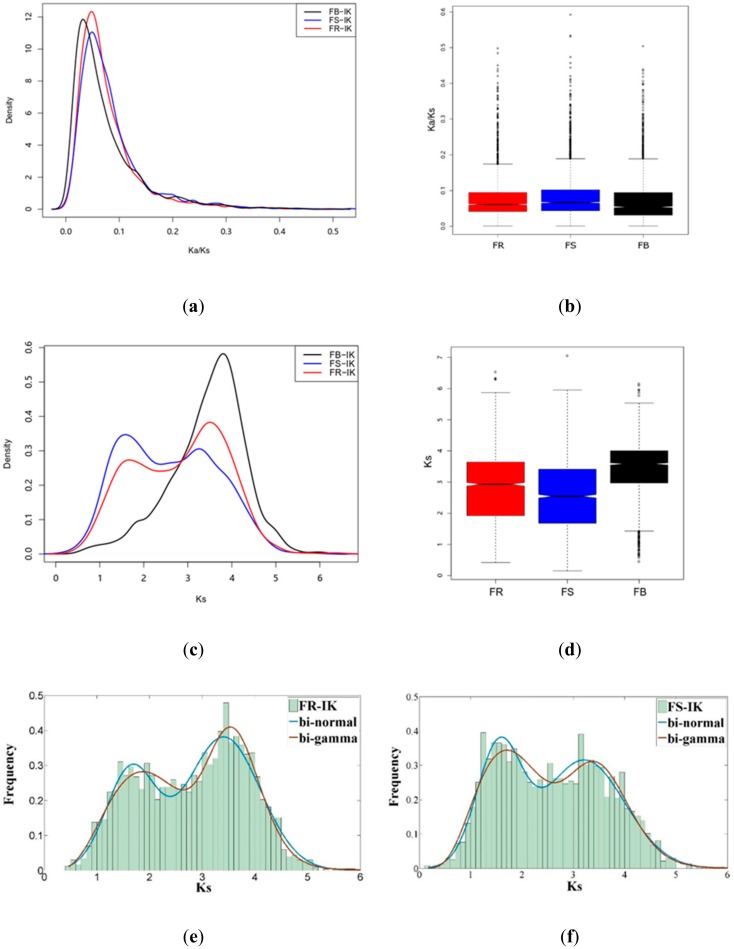
The comparison of ortholog sequence divergence between IK-1^T^ and its phylogenetic neighbors. (**a**) The distribution of *Ka/Ks* values of orthologous genes between IK-1^T^ and its phylogenetic neighbors. (**b**) The comparison of *Ka/Ks* values of orthologous genes between IK-1^T^ and its phylogenetic neighbors by boxplot. (**c**) The distribution of *Ks* values of orthologous genes between IK-1^T^ and its phylogenetic neighbors. (**d**) The comparison of *Ks* values of orthologous genes between IK-1^T^ and its phylogenetic neighbors by boxplot. (**e**) Fitting of Ks frequency of orthologous genes between IK-1^T^ and *F. rivuli*. (**f**) Fitting of Ks frequency of orthologous genes between IK-1^T^ and *F. subsaxonicum*. IK: *F. akiainvivens* IK-1^T^, FB: *F. beibuense* F44-8, FS: *F. subsaxonicum* DSM 21790, FR: *F. rivuli* DSM 21788.

We next investigated the evolution direction of genes in IK-1^T^. We retrieved pairwise orthologs from IK-1^T^-*F. rivuli* (2318 orthologous gene pairs)*,* IK-1^T^-*F. subsaxonicum* (2353 orthologous gene pairs), IK-1^T^-*F. psychrophilum* (1463 orthologous gene pairs), IK-1^T^-*F. johnsoniae* (2016 orthologous gene pairs)*,* IK-1^T^-*F. antarcticum* (psychrotolerant bacterium, 1645 orthologous gene pairs), IK-1^T^-*F. beibuense* (marine bacterium, 2091 orthologous gene pairs), IK-1^T^-*F. sp.* Leaf359 (1892 orthologous gene pairs), IK-1^T^-*F. sp.* root186 (1993 orthologous gene pairs), and IK-1^T^-*F. soli* (1893 orthologous gene pairs). Although these selected *Flavobacterium* species were isolated from different terrestrial or aquatic environments, the peaks of kernel density plots of *Ka/Ks* values (the ratio of nonsynonymous substitution rate (Ka) to synonymous substitution rate (Ks)) were centered less than 0.1, indicating a purifying selection occurred (Figure 5a,b, data not shown for IK-1^T^-*F. psychrophilum*, IK-1^T^-*F. johnsoniae,* IK-1^T^-*F. antarcticum*, IK-1^T^-*F. sp.* Leaf359, IK-1^T^-*F. sp.* root186, and IK-1^T^-*F. soli*).

Intriguingly, the density plots of Ks distributions of orthologous genes between IK-1^T^ and *F. rivuli* or *F. subsaxonicum* displayed two peaks (Figure 5c,d). This bimodal pattern of density plot of *Ks* values of orthologous genes fitted to a bi-normal or bi-gamma distribution (Figure 5e,f). In contrast, the density plots of Ks distributions of pairwise orthologous genes in IK-1^T^-*F. beibuense*, IK-1^T^-*F. psychrophilum,* IK-1^T^-*F. johnsoniae*, or IK-1^T^-*F. antarcticum*, formed a single peak (Figure 5c,d, data not shown for IK-1^T^-*F. psychrophilum*, IK-1^T^-*F. johnsoniae,* IK-1^T^-*F. antarcticum*, IK-1^T^-*F. sp.* Leaf359, IK-1^T^-*F. sp.* root186, and IK-1^T^-*F. soli*). Annotations of the orthologous genes with those lower *Ks* values (*Ks* < 2) identified the genes involved in central dogma, such as 30S and 50S ribosomal proteins, DNA polymerases, and DNA-directed RNA polymerases. These data suggest that more flexible relaxed molecular clock model may apply for bacteria with close phylogenetic relationships. 

### 2.6. Gliding Motility and Type 9 Secretion System in IK-1^T^

To examine the genes absent from the gliding system in the IK-1^T^ genome, we searched the IK-1^T^ genome sequence using both BLASTP and TBLASTN and identified 20 genes involved in gliding motility. We identified the presence of orthologs of 17 gliding genes in IK-1^T^ (Table S7), and found the absence of orthologs of three adhesion-like protein coding genes (*remA*, *remB*, and *sprB*). In addition, the IK-1^T^ genome also encodes type 9 secretion system (T9SS) that are components of gliding motility system (Table S7).

### 2.7. Type 2 Secretion System in IK-1^T^

We next examined the type 2 secretion system in IK-1^T^. IK-1^T^ encodes the core components of a Sec-SRP (signal recognition particle) secretion pathway, containing SecD/F, SecY, YajC, YidC, SecA, FtsY, and SRP54 ffh. We confirmed that IK-1^T^ lacks SecE, SecG, SecM, and SecB by performing BLASTP and TBLASTN analyses. SecY, SecE, and SecG form a transporter complex across the inner membrane, but SecE and SecG are not obligatorily required for their translocation activity [21,22]. In addition, chaperone SecB and secretion monitor SecM are not obligatorily required for the sec-SRP pathway [22]. Thus, IK-1^T^ encodes the essential components of Sec-SRP pathway for a potentially functional type 2 secretion system. 

Peptidases can recycle unfolded or misfolded proteins, remove signal peptides, and function as exotoxins when they are secreted. We used the MEROPS database 10.0 [23] to search for genes encoding peptidase in IK-1^T^, and identified 159 peptidases and their inhibitors grouped in 66 clans. These data reveal that IK-1^T^ encodes fewer peptidases and inhibitors than *F. rivuli* that encodes 177 peptidases and inhibitors [15].

Next, we examined the signal peptides in proteins that may be exported outside IK-1^T^. PrediSi server and SignalP 4.1 server, respectively, predicted 1310 and 754 proteins containing signal peptide cleavage sites. A total of 720 proteins were predicted to contain signal peptide cleavage sites annotated by both programs. Among these proteins, 15 proteins were predicted to contain transmembrane regions, including 10 hypothetical proteins, collagenase precursor, lipid A 1-phosphatase, TonB-dependent receptor plug domain protein, Y_Y_Y domain protein, and carboxylesterase N1hH. 

### 2.8. Eukaryotic-Like Domain Containing Proteins in IK-1^T^

Because bacterial homologues of eukaryotic signaling domains were found in a number of plant pathogenesis-related proteins [24], we further searched the IK-1^T^ genome for eukaryotic-like domains using the effective eukaryotic-like domains (EffectiveELD) database [25]. We predicted 115 proteins containing eukaryotic-like domains (ELD) (Z-score ≥ 4). Thirty-seven proteins containing ELDs also contained T2SS signal peptide, suggesting that they may be secreted by T2SS. Besides GH families and redoxins, ELD proteins encoded in the IK-1^T^ genome include three ankyrin repeat (ANK) containing proteins, four von Willebrand factor type A domain (vWFA) containing proteins, and three major royal jelly proteins (MRJP), which are mainly enriched in symbiont or pathogenic bacteria. 

ANK-containing proteins have been identified in eukaryotes, eukaryotic viruses, and various bacterial taxa [26]. Bacterial ANK-containing proteins can be secreted into host cell, and then hijack host immune responses or modulate global gene transcriptions by mimicking host proteins [26]. IK-1^T^ has three genes encoding ANK-containing proteins. OrthoMCL clustered one IK-1^T^ ANK-containing protein as singleton, and clustered the other two ANK-containing proteins into two different orthologous gene families. The neighbor-joining tree of *Flavobacteria* ANK-containing proteins was constructed using MEGA X based on Dayhoff model and pairwise matching (Figure S7). Both IK-1^T^ ANK-containing proteins were closely clustered with those from *F. rivuli* and *F. subsaxonicum*. A BLASTP search of the NCBI non-redundant sequence database with IK-1^T^ ANK-containing singleton as query revealed significant similarities with the ANK-containing protein from soil bacterium *Solitalea Canadensis*, suggesting that this singleton gene may be acquired by horizontal gene transfer (HGT). 

Von Willebrand factor in metazoans mediates ligand-binding events [27]. Although functions of bacterial vWFA domains remain largely unknown, studies suggest that bacterial vWFA domains may be secreted and have similar ligand and/or protein-binding functions to their eukaryotic homologues [24]. The IK-1^T^ genome encodes four vWFA domain-containing proteins that were clustered into four different orthologous gene families in *Flavobacteria*. Three vWFA domain-containing proteins in IK-1^T^ were clustered with those from *F. rivuli* and *F. subsaxonicum*, but one IK-1^T^ vWFA domain-containing protein was clustered with that from *F. tegetincola* (Figure S8). They contain additional domains such as a BatA domain or a secreted endopeptidase domain that may contribute to IK-1^T^ pathogenesis.

Members of Major Royal Jelly Protein (MRJP) family in insects play diverse roles in physiology, development, and reproductive maturation [28]. MRJP domain-containing proteins were scattered in various bacterial species, suggesting that MRJP family genes with important roles for niche adaptation may be retained in bacterial genomes. IK-1^T^ genome encodes three MRJP genes and OrthoMCL clustered them into three different orthologous gene families in *Flavobacteria*. Two IK-1^T^ MRJP proteins containing a sole MRJP domain were clustered with MRJPs from *F. rivuli* and/or *F. subsaxonicum* (Figure S9). The third IK-1^T^ MRJP protein with an additional N-terminal cupin domain was clustered with the MRJP from *F. hydatis*. 

### 2.9. Quorum Sensing Mechanism

**Figure 6:**
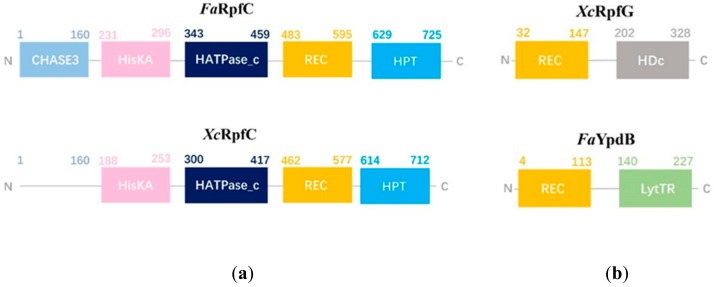
Comparison of domain arrangements of RpfC (**a**) and its cognate response regulator (**b**) in IK-1^T^ and *Xanthomonas campestris*.

Unicellular bacteria can produce and/or respond to signal molecules, named as autoinducers, to cooperate their gene expressions via quorum sensing pathway. However, the quorum sensing system hasn’t been systematically proposed in the genus *Flavobacterium*. To determine the quorum sensing system in IK-1^T^, we performed BLASTP to search the IK-1^T^ proteome using Rpf protein sequences from plant-pathogenic *Xanthomonas campestris* as queries. We identified homologous proteins of PrfB, LysS, RpfG, RpfC, RpfF, RpfB, and PrfA in IK-1^T^. Homologous proteins of RpfE or RpfH were not found by either BLASTP or TBLASTN. Unlike in plant pathogen *Xanthomonas campestris* and *Xylella fastidiosa* where these genes form an *rpf* operon [29], most of these homologous proteins were distributed widely in the IK-1^T^ genome (Table S8). The homologs of RpfG and RpfC still locate adjacent with 212 bp apart and probably form an operon. The homolog of RpfC, *Fa*RpfC, contains HisKA, HATPase_c, REC, and HPT domains in the same order as the RpfC in *X. campestris* (Figure 6a). However, *Fa*RpfC contains an additional N-terminal CHASE3 (Cyclases/Histidine kinases Associated Sensory Extracellular) domain, which is found in transmembrane receptor from bacteria but absent in *X. campestris* RpfC (Figure 6a). Interestingly, the homolog of RpfG with the best e-value is a transcriptional regulatory protein YpdB, *Fa*YpdB. Unlike RpfG which contains a C-terminal diguanylate cyclase domain and regulates bacterial homeostasis of the second messenger c-di-GMP, *Fa*YpdB contains an N-terminal REC domain and a C-terminal LytTR domain, a winged helix-turn-helix (wHTH) which receives the phosphorelay signal to regulate DNA transcription (Figure 6b). This putative two-component sensing signaling pathway, *Fa*RpfC-*Fa*YpdB, is consistent with the lacking of putative diguanylate cyclase or c-di-GMP phosphodiesterase in IK-1^T^, which is confirmed by our BLASTP and TBLASTN analyses. When performing BLASTP using *Fa*RpfC and *Fa*YpdB as the query sequences to search against the NCBI non-redundant sequence database, the ranked top 5 homologous proteins were found in *Chryseobacterium* spp. or *Epilithonimonas* spp., which belong to the Family *Flavobacteriaceae*. This provides evidence that this two-component sensing signaling system may have evolved convergently between IK-1^T^ and species outside of the genus *Flavobacterium*.

## 3. Discussion

Bacteria have been playing important roles in altering environments on Earth for a billion years. For the past decade, massive parallel sequencing technologies and bioinformatic analyses have made tremendous progresses in surveying potential new microbial species. As many as one trillion (10^12^) microbial species have been predicted to exist on Earth, suggesting a much greater biodiversity of microbes than anticipated [30]. Identifications of the isolated cultures of new bacterial species by genome sequencing and physiological studies will help improve our understanding on bacterial ecological roles and the genetic basis of bacterial evolution in the tree of life. Specifically, studies on plant-associated bacteria will shed light on their effects on plant and environmental health. For example, decaying wood inhabiting bacteria help convert organic matters back to usable chemicals, acting as decomposers to re-shape a forest.

More than 130 species in the genus *Flavobacterium* have been identified and 59 of them have their genome sequences available in the NCBI database. By comparing orthologous gene families in *Flavobacteria*, we found that the presence and absence of gene families revealed close relationships between IK-1^T^ and *F. rivuli* or *F. subsaxonicum*, which is consistent with the comparison result of the 16S rRNA gene sequences. *F. rivuli* and *F. subsaxonicum* were isolated from water rivulet of North Germany. However, the detailed analysis of *F. rivuli* suggested that it belongs to the terrestrial clade [5,17]. Synteny analysis revealed extensive synteny relationships exist between the genomes of IK-1^T^ and *F. rivuli*/*F. subsaxonicum*. Although these species were isolated from two spots, far apart on different continents, they showed dramatic conserved genome features. Notably, the density plots of Ks distributions of orthologous genes between IK-1^T^ and *F. rivuli* or *F. subsaxonicum* displayed a bimodal pattern which fitted to a bi-normal or bi-gamma distribution. In our unpublished work, we also found a bi-normal or bi-gamma Ks distribution of orthologous genes in close phylogenetic neighbors in the genus *Luteimonas*. These findings indicate that a more flexible relaxed molecular clock model may apply for bacteria with close phylogenetic relationships. In addition, orthologs of central dogma genes and other house-keeping genes from IK-1^T^ and *F. rivuli* or *F. subsaxonicum* evolve with lower synonymous mutation rates. Taken together, our analyses of gene gain and loss events, conserved genome regions, and synonymous mutation events consistently suggest that *F. rivuli* and *F. subsaxonicum* are the closest phylogenetic neighbors of IK-1^T^. 

Our analyses of gene families of CAZy domains in IK-1^T^ suggest that it belongs to a terrestrial clade of *Flavobacteria*. The nearest phylogenetic neighbor of IK-1^T^, *F. rivuli*, lacks GH10 xylanase and GH115 α-glucuronidase, which act on xylans in the secondary wall of angiosperms and lignified monocots. By contrast, IK-1^T^ encodes one GH10 and one GH115, suggesting that compared to *F. rivuli*, IK-1^T^ obtained GH10 and GH115 to be more active on xylans. In addition, IK-1^T^ encodes one more GH28 than *F. rivuli*, which is involved in degradation of the major structural component of a plant cell wall. IK-1^T^ also encodes four more GH43 endoarabinanases than *F. rivuli*, which degrade important plant polysaccharide arabinan. Taken together, IK-1^T^ has gained extra CAZy gene families during evolution and adapted to plant hosts. 

In the genus *Flavobacterium*, motility system and T9SS share a range of components. Motilities of *F. psychrophilum*, *F. johnsoniae*, and *F. columnare* are based on gliding mechanism. Comparisons of 37 genomes of members of the phylum *Bacteroidetes* defined a core set of orthologs for 11 of the gliding motility genes [31]. In contrast, no gliding motility was observed for IK-1^T^ [14]. The lacks of the three adhesion-like proteins (RemA, RemB, and SprB) were reported in the draft genome sequence of *F. rivuli*, which is also a non-gliding *Flavobacterium* species [15]. These results suggest that like *F. rivuli*, the gliding system encoding genes are maintained in the IK-1^T^ genome but the adhesion-like proteins may be required for gliding. Since the gliding system can serve as a type 9 secretion system, also known as the Por secretion system (PorSS) [31,32,33], the conserved gliding motility genes in IK-1^T^ may function to secrete its T9SS effectors involved in bacteria-host interactions. 

Bacterial secretion systems export proteins or virulence factors out of bacterial membrane to extracellular environment or host cells. Up to date, species harboring T2SS are extracellular pathogens. The proportions of type 2 secretomes in the proteomes of Gram-negative bacteria ranged from 12.6% to 42.4% [34]. Based on the fitted regression equation, secretome percentage = 0.0017 × proteome size + 23.8 [34], the secretome of IK-1^T^ (predicted to be ~30.7%, ~1248 proteins) is consistent with the secretome range in Gram-negative bacteria. Eukaryotic signaling domains shared between eukaryotes and bacteria can be either retained in genomes evolved from last universal common ancestor (LUCA) or acquired by direct HGT [24]. The eukaryotic-like domains in bacteria may have variable functions and mimic eukaryotic counterparts to regulate host physiology. Their important unknown functions restricted the conserved amino acid sequences during evolution. IK-1^T^ genome encodes three or four copies of ANK-, vWFA-, and MRJP-containing proteins, respectively. Sequence comparisons showed that most of the eukaryotic-like domains of IK-1^T^ were closely clustered with orthologous genes from its nearest phylogenetic neighbors *F. rivuli* and *F. subsaxonicum*. One ANK-containing protein is a singleton in *Flavobacteria*, whereas one vWFA- and one MRJP-containing protein was clustered with other *Flavobacteria* species. These suggest that the ANK singleton may be obtained by a unique HGT event, and vWFA- and MRJP-containing proteins were retained from MRCA. Taken together, these proteins containing putative eukaryotic signaling domains may play pivotal roles in bacterial interactions with plant host. Further functional characterizations of these proteins will help determine their roles in bacteria-host interactions.

Plant-associated bacteria use a quorum sensing mechanism to communicate within the community to regulate gene expressions and population behaviors. In this study, we identified a novel putative quorum sensing system, *Fa*RpfC*-Fa*YpdB, in IK-1^T^. *Fa*RpfC with the N-terminal CHASE3 domain represents a new type of the hybrid sensory kinase RpfC. The CHASE3 domain is usually found in MCP (methyl-accepting chemotaxis protein), and the homology model of CHASE3 domain structure suggested a remote similarity to the periplasmic ligand-binding domain of the aspartate chemoreceptor Tar from *Escherichia coli* and *Salmonella enterica* serovar Typhimurium [35,36]. The CHASE3 domain from *Sphingomonas melonis* Fr1 senses heat shock and different salts in the general stress response [37]. Further functional characterization of this novel quorum sensing system will provide insights to the sensing-signaling mechanism in IK-1^T^. 

Taken together, our analyses of IK-1^T^ genomic traits suggest that IK-1^T^ is a plant-associated bacterium and has adapted to terrestrial environment. Using IK-1^T^ as an example, we showed that HGT events occurred and may help IK-1^T^ survive in the plant host. Further functional characterization of IK-1^T^ unique genes will improve our understanding in specific ecological roles and evolution of these new genes. Moreover, large-scale analysis of bacterial orthologous genes may reveal a ubiquitous bimodal distribution of synonymous mutation rate in phylogenetic neighbors. Of note, surveying genomes of environmental microbes will contribute to the gene bank of Earth and provide the genetic basis for evolution analyses.

## 4. Materials and Methods 

### 4.1. Genome Functional Annotation

The genome sequence of IK-1^T^ was annotated in prokka 1.11 [38]. Repeat regions were annotated using RepeatMasker [39]. Protein functions and Gene Ontology (GO) terms were annotated using InterProScan [40]. RNA families were further annotated using Rfam database and perl script rfam_scan [41]. Pathways were annotated using Kyoto Encyclopedia of Genes and Genomes (KEGG) website [42,43,44]. Synteny analysis was carried out using Symap 4.0 [45,46]. Scaffolds above 100 kb were compared in Symap 4.0. Neighbor-joining trees were constructed using MEGA X based on Dayhoff model and pairwise matching [47]. Protein domains were analyzed using SMART (Simple Modular Architecture Research Tool) [48] and NCBI CDD (Conserved Domain Database).

### 4.2. Annotation of Carbohydrate-Active Enzymes and Peptidases

Both the CAZy Analysis Toolkit (http://mothra.ornl.gov/cgi-bin/cat.cgi) and the dbCAN prediction web server (http://csbl.bmb.uga.edu/dbCAN/) were used to annotate genes encoding carbohydrate binding and metabolic enzymes. Only genes were identified by both programs were selected for principle component analysis (PCA) using R. The MEROPS database 10.0 manually curated for peptidase was used to annotate peptidases and the families [23]. 

### 4.3. Clustering of Orthologous Groups 

The Genome sequences of 59 members of *Flavobacterium* spp. were downloaded from NCBI (Table S2) and annotated using Prokka 1.11. Orthologous groups of proteins were identified by filtering all-vs-all BLASTP results and orthoMCL [49]. If the aligned length was less than 80% of the longer length of two protein sequences, the BLASTP result was filtered out. Then if the aligned length was equal to or above 150 amino acids, the BLASTP result with pairwise identity below 30% was filtered out; if the aligned length was less than 150 amino acids, the BLASTP result was filtered by the formula (100 × (0.06 + 4.8 × L^ (−0.32 × (1 + e^–L/1000^)))), in which L stands for the aligned length [50]. Gene accumulation curve was plotted using R package vegan [51]. PCA was carried out using R command ggfortify [52,53]. The percentages of PC1 and PC2 in Figure 2 are 84.9% and 8.49%, respectively. *Flavobacterium* species were clustered using R package cluster. The percentages of PC1 and PC2 in Figure 3b are 15.95% and 4.88%, respectively.

### 4.4. Analysis of Synonymous/Nonsynonymous Substitution Rates

Synonymous substitution rate (Ks) and the ratio of nonsynonymous substitution rate to synonymous substitution rate (Ka/Ks ratio) were calculated by ParaAT and KaKs_Calculator packages [54,55]. Density plots of Ka and *Ks* values were created using R commands. Fitting of Ks distribution frequency was carried out using in-house MATLAB program. 

### 4.5. Analysis of Flavobacterium Akiainvivens IK-1^T^ Secretion System 

PrediSi server (http://www.predisi.de/home.html) and SignalP 4.1 server were used to predict signal peptides in the proteome [56]. EffectiveELD database was used to predicted potential secreted proteins [25]. 

## Supplementary Materials

Supplementary materials can be found at https://www.mdpi.com/1422-0067/20/19/4910/s1.

## Abbreviations

IK-1^T^	*Flavobacterium akiainvivens* IK-1^T^
LUCA	last universal common ancestor
MRCA	most recent common ancestor
HGT	horizontal gene transfer
ELD	eukaryotic-like domains
ANK	ankyrin repeat
vWFA	von Willebrand factor type A domain
MRJP	major royal jelly proteins
