# Supplementary material for: Comparative Genome Analyses Reveal the Genomic Traits and Host Plant Adaptations of Flavobacterium akiainvivens IK-1T
Source: Int J Mol Sci. 2019 Oct 3;20(19):4910. doi: 10.3390/ijms20194910 (PMC6801697; doi:10.3390/ijms20194910)
Supplement: Supplementary file 1 [file ijms-20-04910-s001.zip › Supplements/Supplementary legends.docx]

**Supplementary Figure legends:**

**Supplementary Figure S1 Phylogenetic tree of the 16S rRNA gene sequences in the *Flavobacterium* species.**

**Supplementary Figure S2 Gene Ontology analysis of *F. akiainvivens* IK-1^T^ singletons.**

**Supplementary Figure S3 Syntenic blocks shared between *F. rivuli* and *F. subsaxonicum*.**

**Supplementary Figure S4 Syntenic regions shared between *F. rivuli* and *F. subsaxonicum* visualized by dotplot.**

**Supplementary Figure S5 Syntenic block shared between IK-1^T^ and *E. meningoseptica.***

**Supplementary Figure S6 Syntenic regions shared between IK-1^T^ and *E. meningoseptica* visualized by dotplot.**

**Supplementary Figure S7 Phylogenetic tree of ankyrin repeat (ANK) containing proteins in *Flavobacterium* species.** The first five letters of species or strain names were used to represent the strains.

**Supplementary Figure S8 Phylogenetic tree of von Willebrand factor type A domain containing proteins in *Flavobacterium* species.** The first five letters of species or strain names were used to represent the strains.

**Supplementary Figure S9 Phylogenetic tree of major royal jelly proteins in *Flavobacterium* species.** The first five letters of species or strain names were used to represent the strains.

**Supplementary Table legends:**

**Supplementary Table S1 Annotation summary of *Flavobacterium akiainvivens* IK-1^T^ genome**

**Supplementary Table S2 Summary of repetitive sequences in *Flavobacterium akiainvivens* IK-1^T^ genome**

**Supplementary Table S3 CAZy domains unique in terrestrial *Flavobacteria***

**Supplementary Table S4 *Flavobacterium* spp. analyzed in this study**

**Supplementary Table S5 List of orthologous genes in *Flavobacterium* spp. analyzed in this study**

**Supplementary Table S6 Synteny information in IK-1^T^ and *F. rivuli* or *F. subsaxonicum***

**Supplementary Table S7 Gliding motility-related genes in IK-1^T^**

**Supplementary Table S8 Rpf operon related genes in IK-1^T^**
