# Supplementary figures and images for: Comparative Genome Analyses Reveal the Genomic Traits and Host Plant Adaptations of Flavobacterium akiainvivens IK-1T
Source: Int J Mol Sci. 2019 Oct 3;20(19):4910. doi: 10.3390/ijms20194910 (PMC6801697; doi:10.3390/ijms20194910)

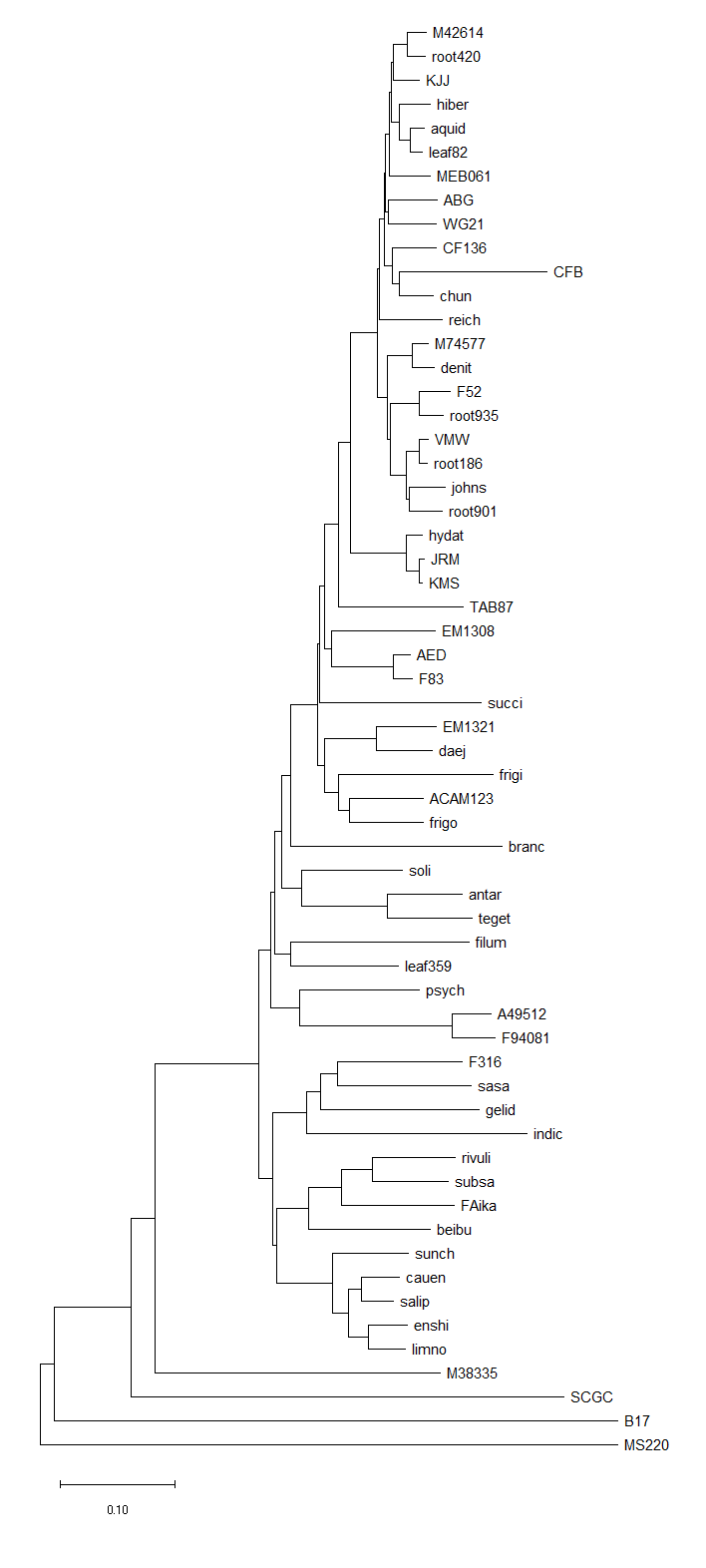

Supplement: Supplementary file 1 [file ijms-20-04910-s001.zip › Supplements/Supplementary Fig S1.tif]

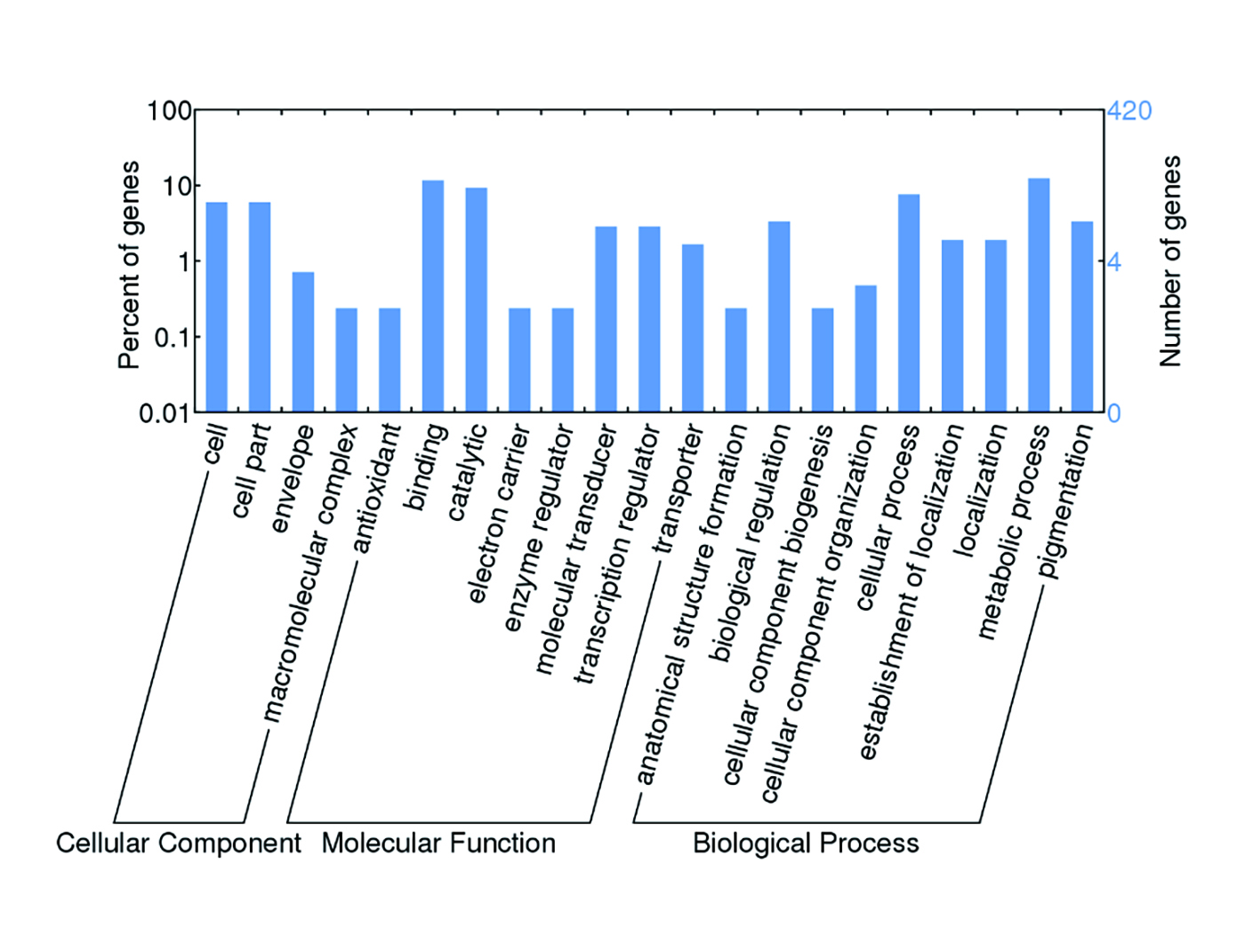

Supplement: Supplementary file 1 [file ijms-20-04910-s001.zip › Supplements/Supplementary Fig S2.jpg]

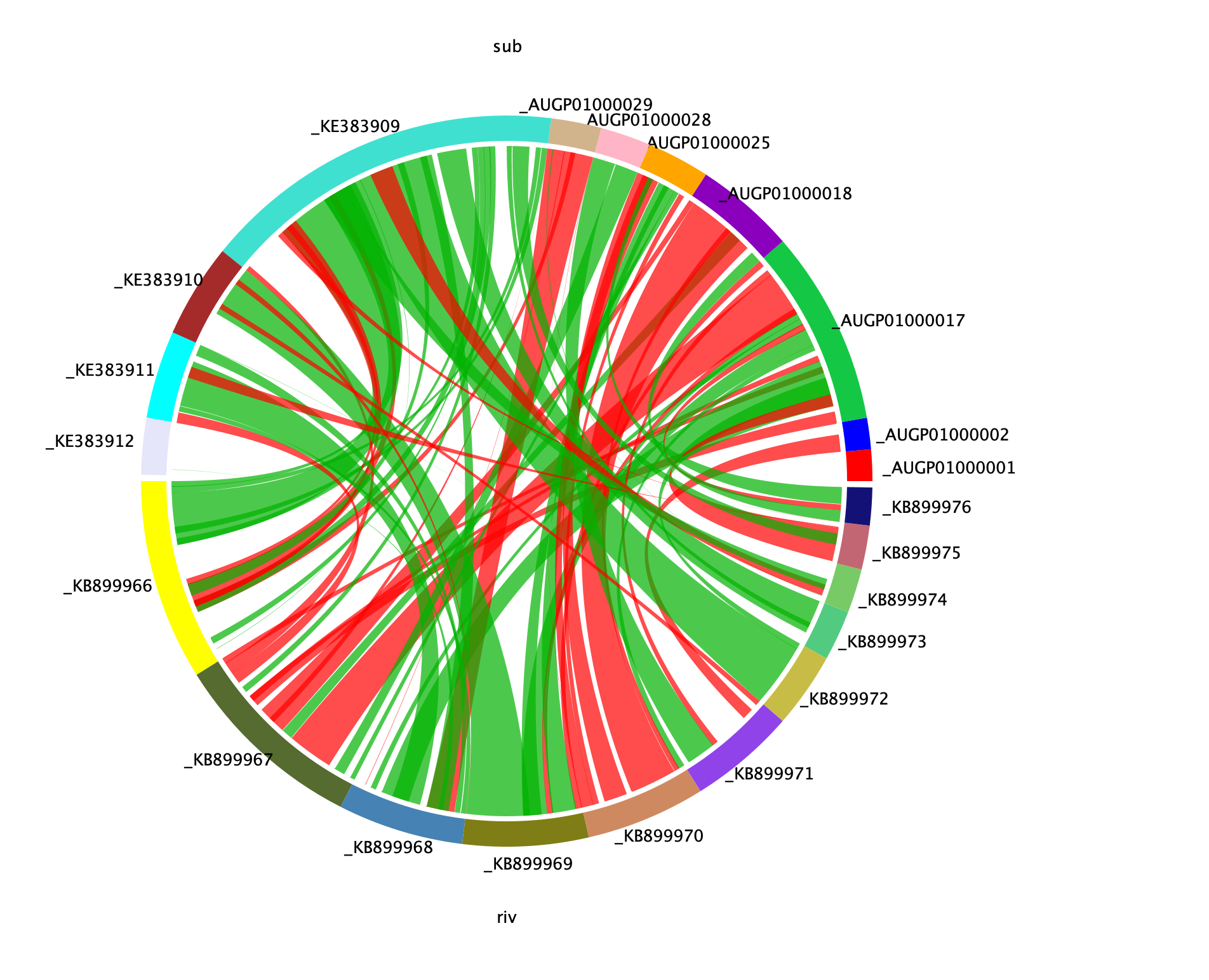

Supplement: Supplementary file 1 [file ijms-20-04910-s001.zip › Supplements/Supplementary Fig S3.png]

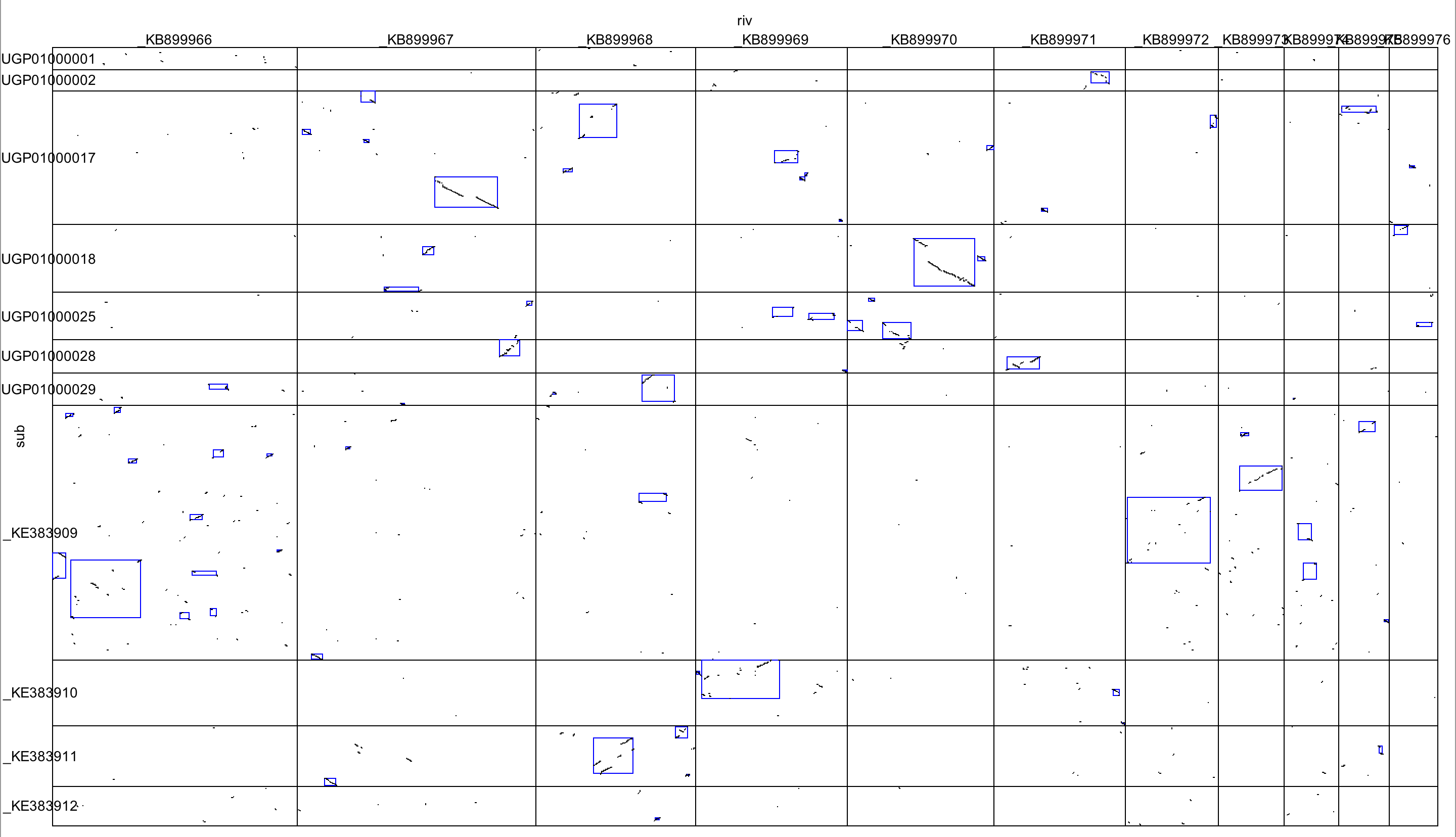

Supplement: Supplementary file 1 [file ijms-20-04910-s001.zip › Supplements/Supplementary Fig S4.png]

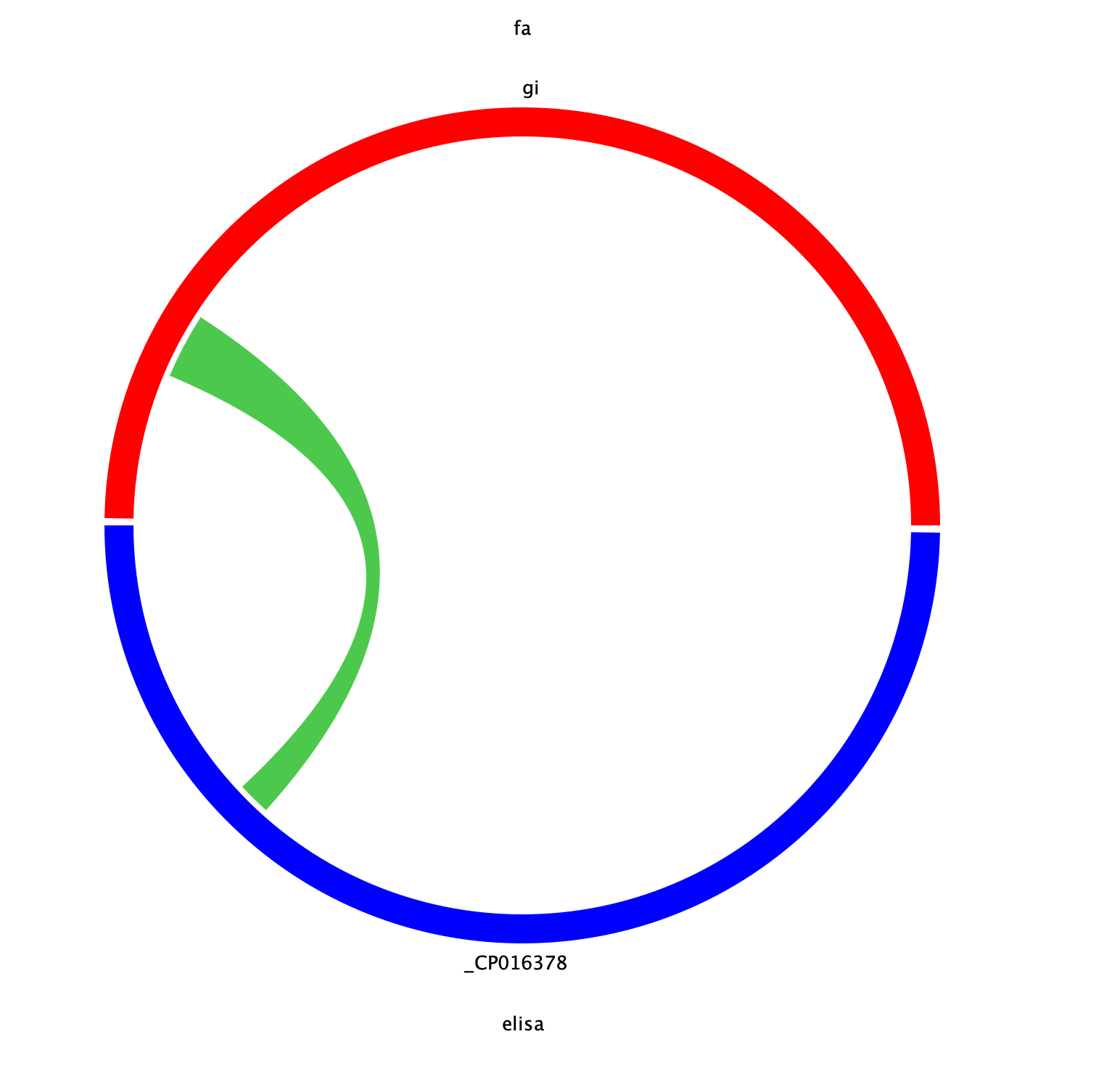

Supplement: Supplementary file 1 [file ijms-20-04910-s001.zip › Supplements/Supplementary Fig S5.png]

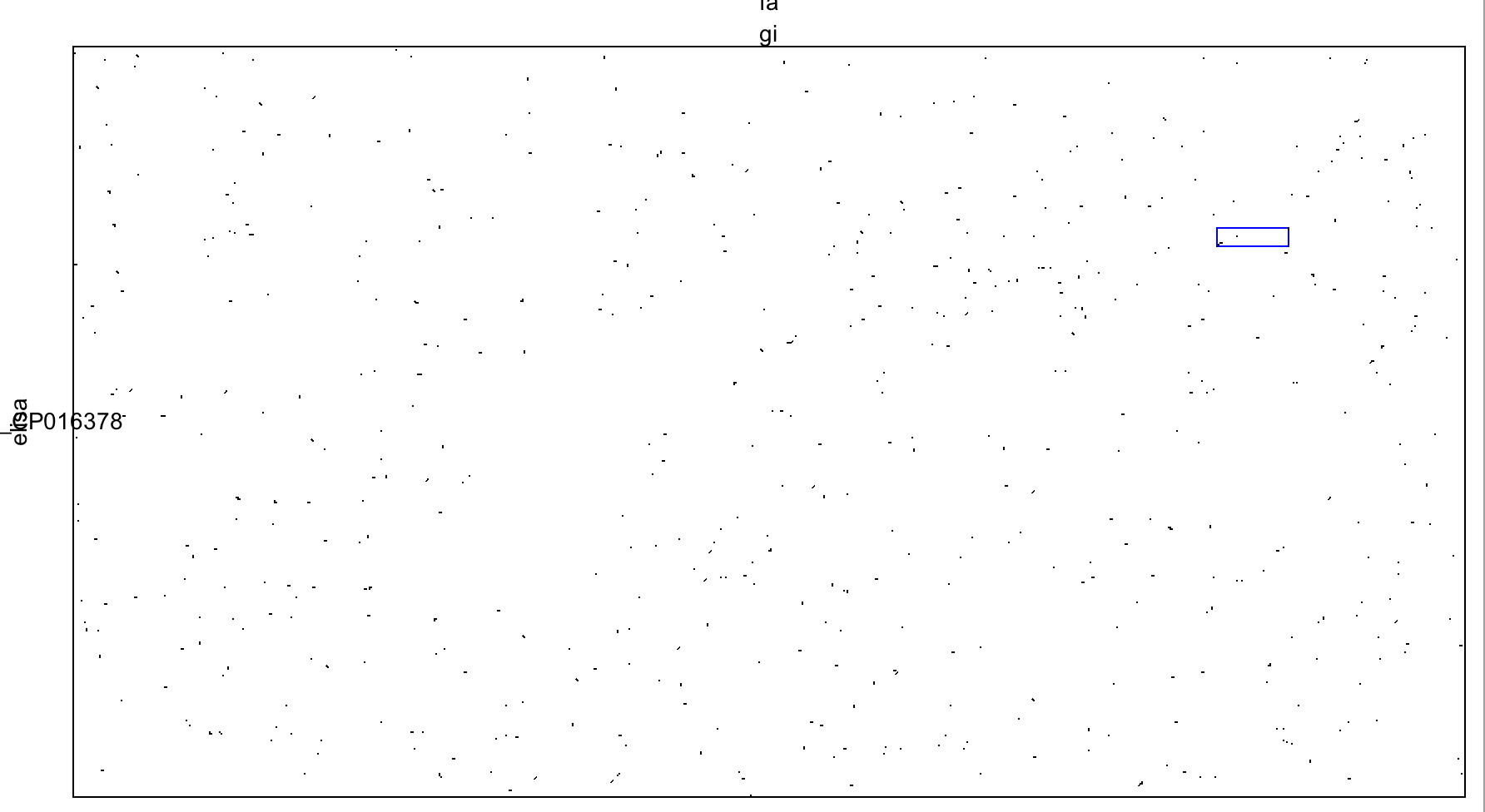

Supplement: Supplementary file 1 [file ijms-20-04910-s001.zip › Supplements/Supplementary Fig S6.png]

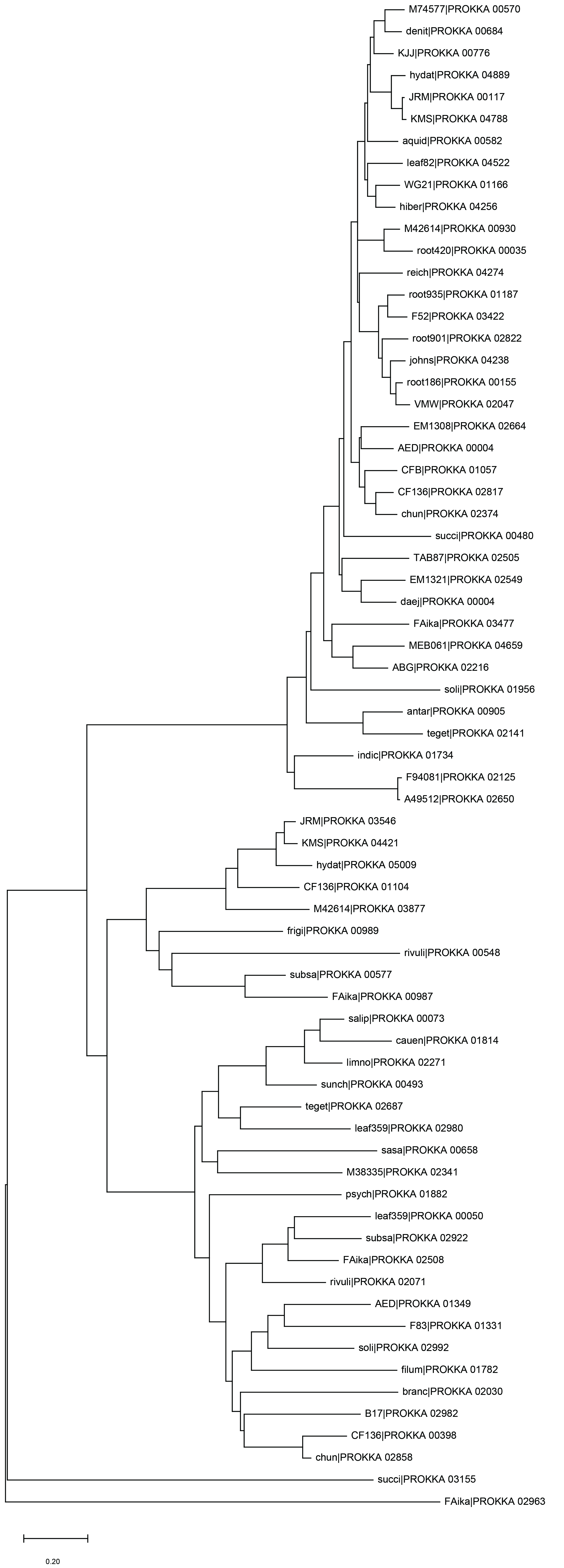

Supplement: Supplementary file 1 [file ijms-20-04910-s001.zip › Supplements/Supplementary Fig S7.jpg]

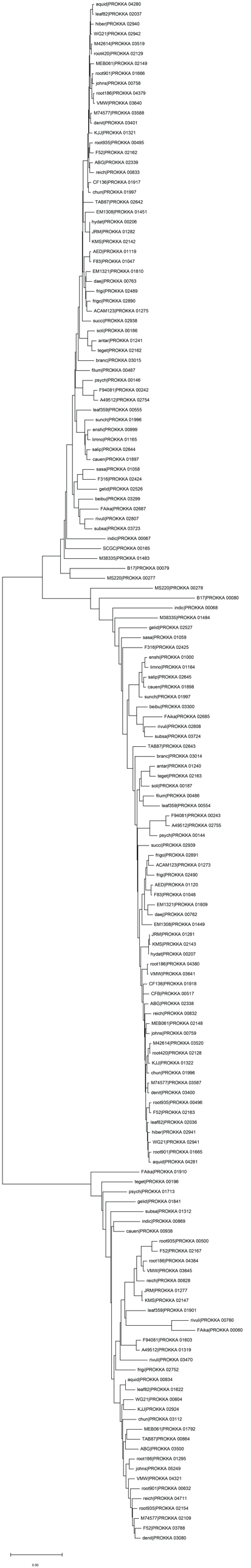

Supplement: Supplementary file 1 [file ijms-20-04910-s001.zip › Supplements/Supplementary Fig S8.jpg]

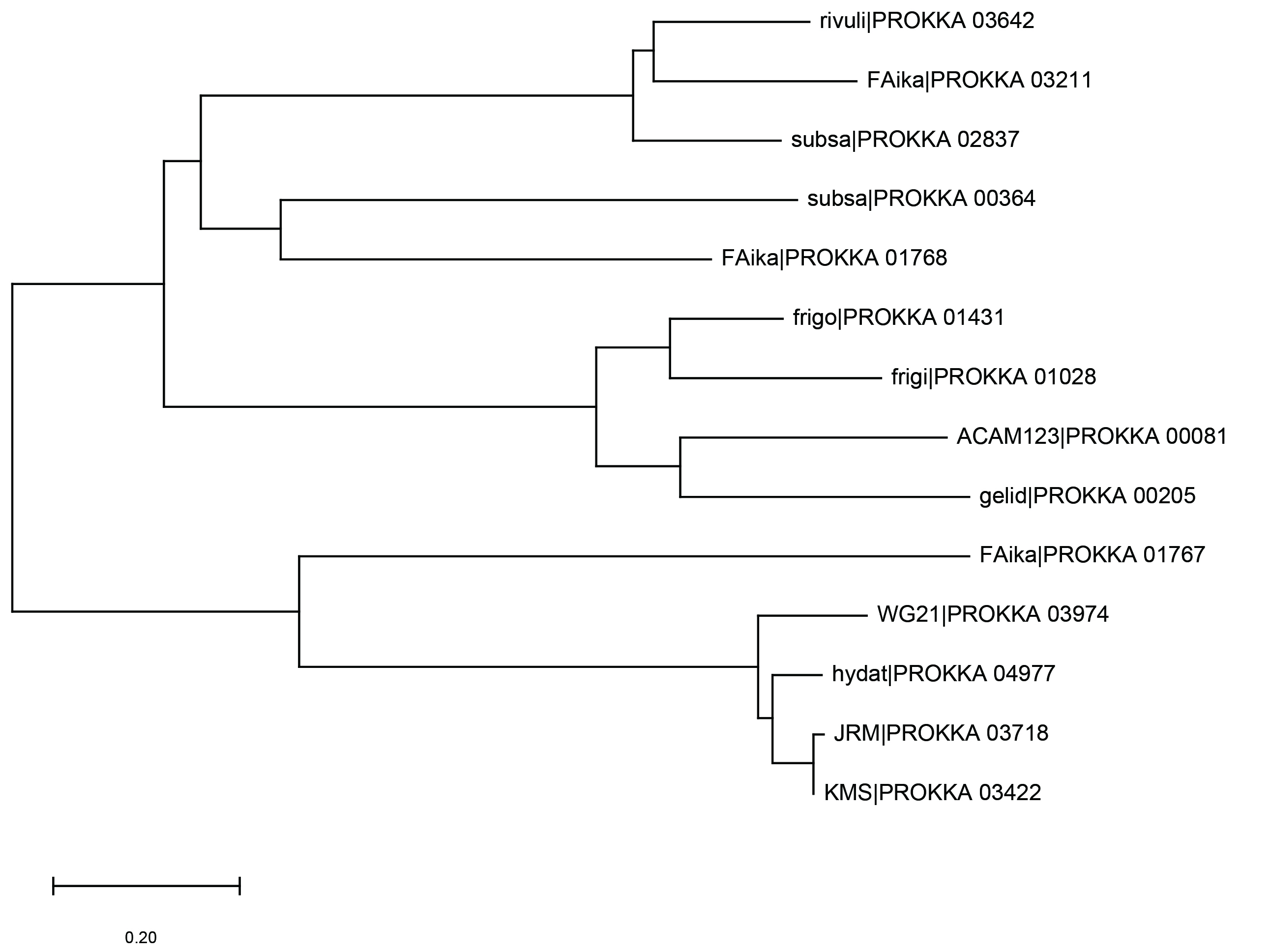

Supplement: Supplementary file 1 [file ijms-20-04910-s001.zip › Supplements/Supplementary Fig S9.jpg]
